# A Hybrid Semantic Knowledge Integration and Sharing Approach for Distributed Smart Environments

**DOI:** 10.3390/s20205918

**Published:** 2020-10-20

**Authors:** Furkh Zeshan, Adnan Ahmad, Abdel-Haleem Abdel-Aty, Fahad Algarni, Emad E. Mahmoud, Ashfaq Ahmad

**Affiliations:** 1 Department of Computer Science, Lahore Campus, COMSATS University Islamabad, Lahore P.O. Box 54000, Pakistan; drzfurkh@cuilahore.edu.pk (F.Z.); adnanahmad@cuilahore.edu.pk (A.A.); ashfaqahmad@cuilahore.edu.pk (A.A.); 2 Department of Physics, College of Sciences, University of Bisha, P.O. Box 344, Bisha 61922, Saudi Arabia; 3 College of Computing and Information Technology, University of Bisha, Bisha, 67714, Saudi Arabia; fahad.alqarni@ub.edu.sa; 4 Department of Mathematics and Statistics, College of Science, Taif University, P.O. Box 11099, Taif 21944, Saudi Arabia; e.mahmoud@tu.edu.sa

**Keywords:** knowledge sharing, quality of service, context-awareness, semantic service matchmaking, smart systems

## Abstract

Distributed systems provide smart functionality to everyday objects with the help of wireless sensors using the internet. Since the last decade, the industry is struggling to develop efficient and intelligent protocols to integrate a huge number of smart objects in distributed computing environments. However, the main challenge for smart and distributed system designers lies in the integration of a large number of heterogeneous components for faster, cheaper, and more efficient functionalities. To deal with this issue, practitioners are using edge computing along with server and desktop technology for the development of smart applications by using Service-Oriented Architecture (SOA) where every smart object offers its functionality as a service, enabling other objects to interact with them dynamically. In order to make such a system, researchers have considered context-awareness and Quality of Service (QoS) attributes of device services. However, context modeling is a complicated task since it could include everything around the applications. Moreover, it is also important to consider non-functional interactions that may have an impact on the behavior of the complete system. In this regard, various research dimensions are explored. However, rich context-aware modeling, QoS, user priorities, grouping, and value type direction along with uncertainty are not considered properly while modeling of incomplete or partial domain knowledge during ontology engineering, resulting in low accuracy of results. In this paper, we present a semantic and logic-based formal framework (hybrid) to find the best service among many candidate services by considering the limitations of existing frameworks. Experimental results of the proposed framework show the improvement of the discovered results.

## 1. Introduction

Dynamic environments are based on different kinds of intelligent objects that work together without human involvement to make their life comfortable [1,2]. Such a smart environment aims to satisfy and to enhance the experience of individuals by replacing the risky work with smart IoT objects. These smart objects while working in a smart environment can provide services ranging from social, economic, environmental, safety to health, and improvement of lifestyle [3]. To connect these objects, while keeping in view their limited resources (battery power, capacity of handling data, and storage, etc.), the academia and industry researchers have proposed some advanced lower power microelectronics along with the Internet Protocol (IP) networking and radio protocols [4] for edge computing. The latest version of Internet Protocol (IPv6), known as the next-generation internet, has the capability to accommodate a large number of smart objects to be connected to the Internet. These technologies are standardized by regulating bodies, led by the Internet Engineering Task Force (IETF), to make them accessible to everyone. One of the objectives of the IETF is to make the smart objects to consume limited power, become IP enabled, and become an integral part of services on the Internet.

On the other hand, the popularity of the Web for applications is to provide standard access to widely available devices such as office computers, laptops, smart telephones, and game consoles to be integrated through the universal platform for the smart applications [5], where every object of smart application can offer its functionality as a service that allows other objects to interact with them dynamically. The functionality offered by such devices may be sensor data, temperature measurement, etc. (in the dynamic environment of health services), referred to as smart object service. Unlike the traditional Web service applications, which are primarily the virtual entity, the smart object services provide real-time data of the physical world. The intelligent system thus can support a more effective decision-making process. However, the key challenge for designers is the integration of a large number of heterogeneous components to manage and share information automatically. Therefore, the architecture and execution of such systems must be consistent with the application requirements, in terms of functionality, execution, reliability, autonomy, and safety, while retaining design and manufacturing costs as low as possible. In this regard, researchers have adapted different knowledge management techniques like case-based reasoning, probabilistic graphical models, artificial neural networks, semantic networks, along with their logical representations [6,7,8]. Among these techniques, semantic networks and logical representations are widely used [9,10,11,12,13,14,15] for managing and sharing of information among smart objects. The wide usage of semantic networks is due to its multiple advantages compared to others. For example, uniform knowledge sharing in the presence of different operating platforms, hardware standards, and languages of smart objects is not addressed effectively [12,15] other than semantic networks. However, in semantic network-based frameworks, the rich context-awareness and Quality of Service (QoS) attributes of smart objects are not considered. Therefore, a framework to manage the complexity and uniform sharing of knowledge between smart heterogeneous system components is needed.

To address these limitations, researchers [9,10,11,13,14,15] have used office computer technology like Transmission Control Protocol/Internet Protocol (TCP/IP) along with Service-Oriented Architecture (SOA) [16]. SOA addresses these issues with the help of semantic web technologies, where the device objects work together and offer their functionality as services (self-described, reusable, and well-defined software components). 

Semantic Web addresses the issues of complexity (communication among hundreds of smart objects) and interoperability (uniform integration of data, obtained from heterogeneous sources) [2,10,17,18,19,20] with the help of ontologies. In fact, ontologies are the abstract model of a defined set of concepts involved in the management and sharing of knowledge in a certain domain. Ontology is a formal way of expressing the device specifications, concepts, and other entities that are assumed to exist in a certain domain interest along with the relations that hold among them [21]. Ontologies ensure interoperability within the applications without misinterpretation of the domain information. However, ontology languages have no capability to model incomplete or partial domain knowledge, whereas the uncertainty exists in almost all phases of ontology engineering. For example, while domain modeling, one might be less interested to know that “is A (class) logically related class B”, but might be more interested to know “how close is A to B” (the degree of similarity).

Traditionally, context-awareness, which is considered as an integral part of smart systems operating in distributed computing environments, is defined as the location, speed, time, and similar physical attributes of an object or a device [22]. However, it should also consider the current state of the environment. Therefore, context modeling is a complicated task as it may include everything around the application. Moreover, the non-functional interactions among edge objects should also be considered in order to understand the behavior of the complete system. Particularly, in a scenario where, if more than one smart object is providing similar or identical functional services to other objects upon their request, in such scenarios, the biggest challenge for requesting an object would be to find the best service while considering context-awareness and QoS attributes. 

In this regard, researchers have done a lot of work [23,24,25,26,27]. However, QoS metrics, user priorities, value type direction, service-ranking, and rich context-awareness along with the uncertainty of incomplete domain knowledge modeling for smart systems operating in distributed computing environments are not considered properly, resulting in low accuracy of results. 

Therefore, in this paper,

- A logic-based semantic hybrid framework upon the above-discussed limitations is proposed that uses Semantic Web technologies to build dynamic context and QoS models for the sharing of knowledge.
- The proposed framework provides a dynamic environment for the sharing of domain information among devices. The framework uses an ontology-based (QoS, application, and context-awareness) service discovery mechanism for modeling the request and a service-matching algorithm.
- In order to verify the effectiveness of the proposed service discovery mechanism, we have statistically compared it with one of the best works available in literature along with the comparative evaluation.
- Results show that the proposed framework has better performance as compared to the existing work.

The rest of the paper is organized as follows: related work is reviewed in Section 2; in Section 3, a mechanism on service discovery by semantic matching is provided; Section 4 presents the evaluation process followed by Section 5 for results and discussion and, finally, Section 6 concludes the paper.

## 2. Related Work

A number of dynamic service discovery approaches are already proposed by different researchers [23,24,25,26,27], considering context-awareness and quality of service criteria of the smart environments. The literature on service discovery approaches is quite large. So, the paper selection criteria are defined as:

- Paper publication duration 2000 to 2020
- Application methods defined clearly
- Paper titles, abstract, and the keywords related to the topic

The exclusion criteria are:

- Unclear, non-indexed papers
- Journal editorials
- Short notes of keynote speakers
- Repeating papers

In order to understand the limitations and their effects in the smart environments, different semantic service discovery approaches are investigated and analyzed in this section. In this regard, we have divided the literature into two categories. The first category is based on the service discovery frameworks that are highly influenced by QoS, while the other is influenced by context-awareness. Details of both categories are given below.

### 2.1. QoS-Based Service Discovery Frameworks

Baocai’s Framework [23] is based on the QoS ontology for the automatic discovery of services. The ontology of the framework is described in the OWL to solve the issue of interoperability. The description of QoS ontology can help the service applicant in finding the best available service. However, neither the discovery of the service nor the services operations are organized and discussed in detail. MA and colleagues [28] have proposed a QoS-based framework for advertising and the discovery of semantic web services. The framework uses the description logic reasoning to investigate the compatibility of demand and service concepts. However, the framework has not been studied for its performance nor has it been studied in detail for parameters of QoS. Chua’s framework [29] performs service discovery based on the QoS attributes in a visual context. The framework uses QoS criteria along with the graphical user interface (GUI) pattern to model the user request in a visual context to discover services by taking into account the user priorities. However, the existing design cannot be implemented because the represented adaptability mechanism does not consider rich QoS attributes and service discovery and selection process. Another framework proposed by Ayadi et al. [30] uses canonical web service models for flexible matching of services. In this framework, the deductive technique is used to resolve and relax query constraints before seeking services. The proposed service selection and ranking process takes into account the weights assigned by the user while considering the candidate services. However, the framework was not evaluated for efficiency, and the ranking process of services was not clearly defined. In addition, Zhao’s framework [31] can congregate, consolidate, and store data from various QoS Web services. The author had performed an experiment to show the flexibility of the framework. They have used Web Services Description Language (WSDL) along with Java data structures to collect QoS data from web services. However, no mechanism has been proposed to effectively and completely assemble QoS data in the distributed environment. Whereas, the framework proposed by Li et al. [32] uses QoS and Web Service Modeling Ontology (WSMO) for Web service in the execution environment (based on the core and expansion parts). Services are assembled functionally in the core part while keeping in view the QoS points and the feedback is sent back to the service user. Nevertheless, the framework does not provide the context description of the relationship between the service provider and the service applicant. Similarly, a framework proposed by Cao et al. [33] uses QoS-aware service recommendation and factorization-based relational topic model for IoT Mashup application. Authors have used risk terrain modeling (RTM) to model and predict the relationships among Mashup and services by mining the latent topics. The framework recommends top N web application programming interface (API) for targeting IoT Mashup applications, but the framework neither considers user priorities nor returns the categorized list of APIs.

### 2.2. Context-Aware Services Discovery Frameworks

Framework based on the TPSSMA algorithm is proposed by Zhu [34] to select and categorize services by considering user’s functional and non-functional requirements. However, the framework is not properly implemented and evaluated for accuracy and performance. Moreover, the framework returns the partially ordered list of services that correspond to the functional requirements of the user. Another framework, proposed by Jimenez-Molina et al. [35] provides support to chronic patients, medical staff, and other relevant participants during the primary health attention process. To treat chronic patients, the framework considers medical organizational requirements. The authors have used Business Process Management (BPM) notation to infer requirements from any support system. However, the framework does not analyze or include existing medical domain ontologies for developing an ontology through the alignment method. The framework proposed by Arabshian [36] has been developed for personalized ontology-based context-aware research. The authors had used the extended ontology developed by Newman [37] to draw the knowledge from a hybrid hierarchical peer-to-peer network. However, the framework does not consider location, timeliness, role, and resources during the discovery of the services. Likewise, Ahmed et al. [38] have proposed a framework for ubiquitous environments to manage the context. The framework uses Resource Description Framework (RDF) and RDF(s) languages for the semantic representation of the context. With this framework, developers can express the context without a unified context model. Although their mechanism maintains a log of context changes to minimize the cost of communication, the impact of change in the context model triggered by the developer was not considered at all. Further, SOCOM [39] explores sensor characteristics to provide relationships between sensors and the context. It described the general knowledge of the real world and its usage in a context-aware middleware. Moreover, the framework [40] proposed by Santos discover and compose dynamic services based on the user-defined goal. For this purpose, the author has used context-awareness along with the requester’s requirements to define goals and to reduce user involvement. The framework is based on domain ontologies (assuming that experts have defined them clearly) to provide a solid foundation (semantically) to the terms used in the framework for defining and achieving the goal. Furthermore, the framework proposed by Jia et al. [41] aggregate IoT services dynamically, based on real-time sensing of information. In order to achieve the goal, the author created service combination categories according to user needs and QoS parameters (functional and non-functional requirements). They used semantic technology and dynamic programming for creating service category combinations and quality of service parameters, defined in terms of requirements for selecting the best service among candidate services. However, the framework does not consider user priorities and context-awareness context modeling.

### 2.3. Comparative Evaluation of Discovery Frameworks 

Comparative evaluation is used for analyzing similarities and differences in a system. Comparative evaluation criteria are always interpreted in the context of some requirements that are sometimes based on research and/or theoretical frameworks.

In this regard, Zeshan et al. [9,42,43] have proposed a framework that provides criteria to compare information systems. The criteria are classified into two categories: General Requirements (Table 1) and Dynamic Requirements (Table 2). This is a minimal set of criteria requirements that must be addressed by every successful real-time system.

The development of successful distributed smart systems depends on the correct requirements. In this regard, the minimal set of requirements for smart systems are defined in Table 2.

In Table 3, the work reviewed in Section 2.1 and Section 2.2 is compared and analyzed on the criteria requirements defined in Table 1 and Table 2. The main objective of this comparison is to evaluate the usefulness of existing approaches and to estimate the degree of effectiveness in the discovery ranking and selection of smart services.

In this section, a large number of research works are investigated and analyzed with regard to their scope and functionality. We have compared different service discovery mechanisms (Table 3) based on the defined criteria requirements. By analyzing the data of Table 3, it is observed that the work proposed by Bandara et al. [44] and Kritikos and Plexousakis [45] are mature works as both fulfills eight requirements each. Whereas, the work proposed by Bandara et al. [44] mostly fulfills the dynamic requirements, while the work of Kritikos and Plexousakis [45] mostly fulfills the general requirements.

Although the above-discussed work has addressed a lot of issues of efficient and effective discovery of services, few issues remain that need the attention of researchers for the effective discovery of device services. The main limitations observed in these approaches are a theoretical model along with the lack of richness in context modeling, the absence of grouping, value type direction, group prioritization, and timeliness, etc. These limitations, if not considered properly, may result in the difficulty to find the best service among many functionally similar services.

## 3. Service Discovery by Semantic Matching

Due to heterogeneity and domain-specific requirements, smart systems need a dynamic object discovery mechanism, which should consider the objects’ semantic descriptions in terms of their attributes (QoS and context), location, and access methods, as a service. Depending on the application, discovery can be a stand-alone service or integrated with the entity. In this context, several service discovery approaches have been proposed [45,54,55,56,57], which consists of identifying a similarity degree between concepts semantically. Particularly, researchers kept much of their focus on word semantics and sentence semantics. However, the calculation of semantic similarity of services is not clearly defined due to the complexity of dynamic environments where devices have massive information, which makes it hard to measure their semantic similarity.

**Figure 1:**
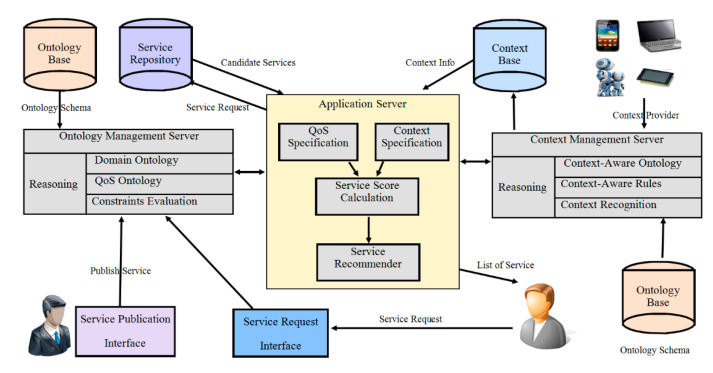
Semantic service discovery framework in a dynamic environment.

The proposed service discovery framework, as depicted in Figure 1, consists of a standard service search-and-discovery mechanism. The framework consists of a service repository and two servers for managing ontology and knowledge bases. This framework addresses many limitations of techniques (discussed in the literature) for describing and discovering qualified services. It is supported by an algorithm to select the best services from many available functional similar services. Context Knowledge Base stores all contexts of devices along with the states of entities. In the services profiling process, the QoS information along with context specification of device services are provided to algorithms from the service repository and the context knowledge base. The following sections present the process for calculating the semantic similarity of services (advertisements and the request attributes).

### 3.1. Score Calculation Using Context Matching

In order to reduce human involvement while composing service to achieve the maximum degree of automaticity, researchers [21,55,58] have considered context-awareness in service-based solution applications from a couple of decades. Moreover, to decrease the issues of interoperability, they have used context-aware ontologies (machine-readable contents). However, to achieve the said goal, different researchers have adopted different methods but none of them clearly demonstrate how to use their proposed technique to select the best service from a number of candidate services available for composition. While using the taxonomy information for score calculation, some rules for semantic similarity score calculation are defined below.

ContxMatching(SrvOffer, SrvDemand) = (SrvOffer = SrvDemand) ˅ (SrvOffer ⊆ SrvDemand) ˅ (SrvDemand ⊆ SrvOffer)

where

(SrvOffer = SrvDemand) = (SrvOffer.IsNumeric ˄ SrvDemand.IsNumeric) ˄ (SrvOffer.Value = SrvDemand.Value)

(SrvDemand ⊆ SrvOffer) = (SrvOffer. IsNameConcept ˄ SrvDemand. IsNameConcept) ˄ (SrvOffer.Level ≤ SrvDemand.Level)

(SrvOffer ⊆ SrvDemand) = (SrvOffer. IsNameConcept ˄ SrvDemand. IsNameConcept) ˄ (SrvOffer.Level ≥ SrvDemand.Level)(1)

To determine the semantic deviation among demand and offer concepts of service, the following rules are used.

Level_SrvOffer = ∑ Level(SrvOffer)

If

Level(SrvDemand) ˂ Level(SrvOffer)(2)

Schar_Atrib = (CountAttribs(SrvDemand)/CountAttribs(SrvOffer)

where *CountAttribs(SrvOffer)* defines the total number of attributes of class Offer. Aziz et al. [20] has used the description logic technique along with the ontology for describing services. Whereas, for computing semantic similarity among services, he has used description logic reasoner, but the proposed technique has not used priority and group priority techniques, resulting in compromised accuracy of matching results. Based on these limitations, below (Equation (3)) is a proposed method for considering user-assigned priorities.

(3) $$ContextScore\;=\overset{charAttrib}{\underset{n=1}{\sum }}S_{n}\left(\mathrm{charAttrib}*W_{D}\mathrm{charAttrib}\right)\;$$

where *W_D_*(charAttrib) is the weight of character attributes of user demand.

### 3.2. Score Calculation Using QoS Metric Matching

One of the biggest challenges for SOA-based service discovery techniques is to ensure the quality of service (QoS) in dynamic environments [59,60]. In this regard, considerable research [23,25,26] has been done to support dynamic attributes of services ranging from expanding Universal Description, Discovery, and Integration (UDDI) to register dynamic attributes of services to store and manipulate dynamic attributes. However, the enhanced techniques do not involve QoS factors that can affect dynamic score calculation, resulting in compromised service selection. Therefore, to address the problem, user-defined priorities, group priorities along with the context-awareness and value type direction has been considered in this study. Whereas, to calculate the semantic similarity between offer and demand concepts, the rules defined by Cortes [61] and modified by Kritikos [45] are used while keeping in mind the OWL descriptions of QoS metric. The equivalence rules proposed by Cortes [61] are mathematically expressed below.

conformance (O_i_, D) ⇔ S (P^O^_i_ ˄ ¬ P^D^) = false

⇔ S (P^O^_i_ ˄ ¬ (C^D^_1_ ˄ C^D^_2_ ˄…˄ C^D^_2M_)) = false

⇔ S (P^O^_i_ ˄ (¬ C^D^_1_ ˅ ¬ C^D^_2_ ˅…˅ ¬ C^D^_2M_)) = false

⇔ S ((P^O^_i_ ˄ ¬ C^D^_1_) ˅ (P^O^_i_ ˄ ¬ C^D^_2_) ˅…˅ (P^O^_i_ ˄ ¬ C^D^_2M_)) = false

⇔ (S (P^O^_i_ ˄ ¬ C^D^_1_) = false) ˄ (S (P^O^_i_ ˄ ¬ C^D^_2_) = false) ˄…˄ (S (P^O^_i_ ˄ ¬ C^D^_2M_) = false)

Equivalence rules take offer and demand as input and try to solve the constraints defined over the set of input. Conformance metric returns true if the problem is resolved and false otherwise by using function S. However, to calculate the closeness between two QoS metric descriptions, the rules proposed by Kritikos as OWL-Q [45] are extended as follows.

QoSMatching (O, D) ⇔ S (O_p_ ¬ D_p_) ˄ Match(O_M_, D_M_)

S (O_p_ ¬ D_p_) <= D_p_ ˄ (O_p_^i^ ˅ O_p_^i+1^ ˅ O_p_^i+1^... ˅ O_p_^i+k^) = true

Match (M1, M2) <= RM (M1) ˄ RM (M2) ˄ sm(M1, M2)

sm(M1, M2) <= svm (M1.scale, M2.scale, M1.type, M2.type, M1.valuetypedirection, M2.valuetypedirection) ˄ M1.object = M2.object ˄ M1.measures = M2.measures (4)

In the above given rules, *M1* and *M2* are two resource metrics where *svm(M1.scale,M2.scale,M1.type,M2.type, M1.valuetypedirection, M2.valuetypedirection)* is a method which compares the scale and type of both objects. This method solves the constraints defined over the metric M1 and M2 and returns true if the solution exists, or false otherwise. Value type direction means what (higher or lower) values are preferable. In case of higher values are preferable, the following proposed formula (Equation (5)) can be used.

(5) $$S_{Attrib}\;=\;1\;-\;\frac{\left(Demand.value\;-\;\;Offer.value\right)}{Demand.value}\;$$

However, in case of lower values are preferable, the following proposed formula (Equation (6)) can be used.

(6) $$S_{Attrib}=\frac{1}{1\;-\;\;\frac{\left(Demand.value\;-\;Offer.value\right)}{Demand.value}}\;$$

Whereas, user-defined priorities can be calculated by using Equation (7).

(7) $$QoSScore=\overset{numAttrib}{\underset{j=1}{\sum }}S_{j}\left(\mathrm{numAttrib}*W_{D}\right)\;$$

### 3.3. Service Ranking

Service ranking is the procedure of rating the candidate services according to the user-defined criteria (context-awareness and the numerical QoS values). The mobility and the heterogeneity of devices operating in a dynamic environment may lead to the ever-changing of available services. Therefore, the service discovery process should return the best suitable service to the requester as per his/her requirements while keeping track of all candidate services in the descending order in requirements. The proposed technique performs this task by using the following formula (Equation (8)).

(8) $$TotalScore\;=\;\overset{n}{\underset{1}{\sum }}\left(Score_{QoS}\;*\;W_{D}\left(QoS\right)\right)\;\;+\;\overset{n}{\underset{1}{\sum }}\left(Score_{Context}\;*\;W_{D}\left(Context\right)\right)\;\;$$

After score calculation, the next step is to rank them based on their final score and the user-defined criteria. Therefore, in order to achieve this goal, the method defined by Kritikos [45] along with the expert judgment is used. However, before defining the ranking criteria, a focus group with experts was conducted and, upon consensus, the following rules were defined.

If (SrvOffer_TotalScore_ ≥ SrvDemand_TotalScore_) then Super Match

If (SrvOffer_TotalScore_ ≥ SrvDemand_TotalScore_ × UpperDefinedValue) ˄ (SrvOffer_TotalScore_ < SrvDemand_TotalScore_) then Good Partial Match

If (SrvOffer_TotalScore_ ≥ SrvDemand_TotalScore_ × LowerDefinedValue) ˄ (SrvOffer_TotalScore_ < SrvDemand_TotalScore_ * UpperDefinedValue) then Partial Match

If (SrvOffer_TotalScore_ < SrvDemand_TotalScore_ × LowerDefinedValue) then Fail Match. (9)

### 3.4. Service Ranking Algorithm

To find a service that addresses the maximum number of user-defined criteria requirements from many similar services is the key challenge in the device services discovery [62,63]. Services are usually defined in their functional parameters, whereas QoS parameters are used to define the behavior of the service. In this regard, context awareness as additional criteria can be used to select the best service. Therefore, QoS and context awareness as a main service selection tool are used in the proposed approach to select the best service. Hence, an algorithm (Algorithm 1) for the discovery and selection of the best service among many functional similar services based on the limitations of the existing work [44,45] is proposed below.
**Algorithm 1.** Services Discovery Ranking and Categorization.1: QoSMatchingAndScoreCalculation (SrvOffer, SrvDemand)2: Int QMetricsSrvDemand = countMetrics (SrvDemand)3: Int QMetricsSrvOffer = countMetrics (SrvOffer)4: Doble TotalQoSScore[num], score5: For(I = 0, I < QMetricsSrvOffer, I = I + 1) 5.1: For(J = 0, J < QMetricsSrvDemand, J = J + 1)  5.1.1: If (I.value typedirection = J.value typedirection AND I.value typedirection = up)   5.1.1.1: If (I.valuetypedirection = J.I.valuetypedirection = up)   5.1.1.2: Score = (1 − (SrvDemand.value − SrvOffer.value)/SrvDemand.value)) * MetricPriority  5.1.2: Else   5.1.2.1: Score = 1/(1 − (SrvDemand.value − SrvOffer.value)/SrvDemand.value)) * MetricPriority6: QoSScore[j] = Score7: Score = 08: End of QoSMatchingAndScoreCalculation (SrvOffer, SrvDemand)9: ContextMatchingAndScoreCalculation (SrvOffer, SrvDemand)10: Int ContextMetricsD = countMetrics (SrvDemand)11: Int ContextMetricsSrvOffer = countMetrics (SrvOffer)12: Doble TotalContextScore[num], score13: For (I = 0, I < ContextMetricsSrvOffer, I = I + 1) 13.1: For (J = 0, J < ContextMetricsD, J = J + 1)  13.1.1: If (Level _(SrvDemand.Context)_ ≤ Level _(SrvOffer.Context)_)   13.1.1.1: Score = MetricPriority  13.1.2: Else   13.1.2.1: Score = Level _(SrvOffer.Context)_/Level _(SrvDemand.Context)_ * MetricPriority14: QoSScore[j] = Score15: Score = 016: End of ContextMatchingAndScoreCalculation (SrvOffer, SrvDemand)17: ServiceRanking(ContextScore[], QoSScore[], int i, int j)18: TotalScore = Sum(ContextScore[i]) * groupPriority19: TotalScore = TotalScore + Sum(QoSScore[j]) * groupPriority20: If (SrvOffer_(TotalScore)_ ≤ SrvDemand_(TotalScore)_) 20.1: AddIn(SuperMatchList[],SrvOffer)21: If (SrvOffer_(TotalScore)_ ≥ SrvDemand_(TotalScore)_ * UpperDefinedValue) 21.1: AddIn(GoodPartialMatchList[],SrvOffer)22: If (SrvOffer_(TotalScore)_ ≥ SrvDemand_(TotalScore)_ * LowerDefinedValue) 22.1: AddIn(PartialMatchList[],SrvOffer) 22.2: Else  22.2.1: AddIn(FailMatchList[],SrvOffer)23: End of ServiceRanking(ContextScore[], QoSScore[], int i, int j)

The above algorithm calculates service scores and ranks them in two steps. In the first step, QoS and the context-aware score is calculated while considering the user-defined priorities. Whereas in the second step, group priorities are considered before computing the final score and ranking the services, Equations (1)–(7) are used.

The framework proposed in this section is based on the limitations of techniques discussed in the literature review section for describing and discovering smart device services. It is supported by an algorithm to select the best services from many available functional similar services. This algorithm uses equations defined in the text to calculate the semantic similarity of services. Upon users’ request submission, the proposed framework computes service scores in two steps. In the first step, the similarity of context attributes is measured using Equations (1)–(3). While, in the second step, the score of QoS attributes is measured using Equations (4)–(7). Equation (8) calculates the final score of service by considering the user-defined priorities, whereas, Equation (9) categorizes the discovered services according to their score.

## 4. Evaluation

**Figure 2:**
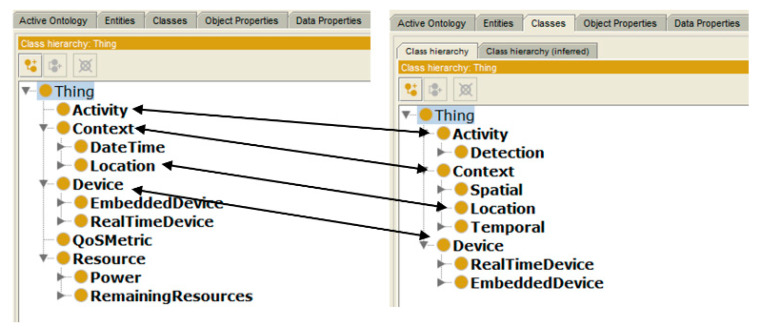
Context and mobile robot ontologies mapping.

**Figure 3:**
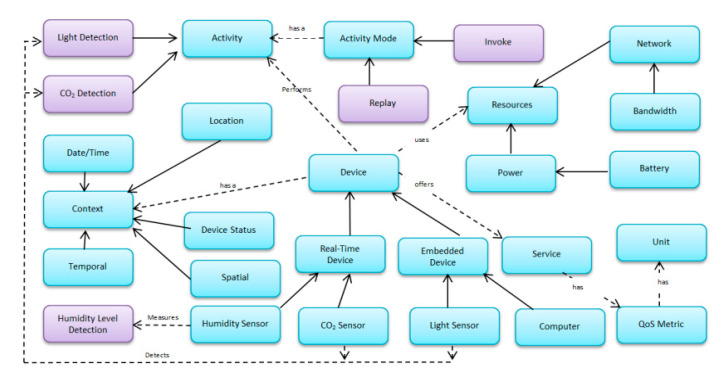
Unified Modeling Language (UML) Meta-model for autonomous mobile robot ontology.

Before applying a system in a real-world scenario, it is required to test it thoroughly to ensure that the system will perform well without major problems. In this regard, an experimental evaluation of the proposed technique is performed using a case study of a hospital’s dynamic environment. Nowadays, many hospitals are equipped with devices like sensors, where the integration of device features to make a service is a challenging task. In such a dynamic environment, services may allow better caring of patients (room/ward temperature, humidity level, as well as differential pressure) by monitoring conditions to restrict the bacteria to promote in the rooms requiring constant supervision. Such services not only ensure the comfort of patients but also guarantee the good health of people working in the hospital. If these conditions are not controlled, then the risk of the spread of harmful bacteria among health care professionals along with patients increases, resulting in spread of disease. However, it is a big challenge in the healthcare environment to manage multiple connected devices and their interoperability. In order to handle such issues, ontologies can be used. Therefore, context-aware ontology [43] along with application ontologies [54] are used to model requests to discover services. However, for creating the service request, both ontologies were merged as presented in Figure 2. In this regard, Protégé editor is used that works on the idea of mapping and merging of ontological concepts. To create a new single extended knowledge sharing ontology, the editor takes a union of the terms like classes, slots, facets, and instances, followed by mapping and alignment processes. Since ontologies are merged using protégé editor, therefore, to make sure that sharing and exchange of complex information messages are well understood, a meta-model that can facilitate semantic interoperation between heterogeneous information resources is defined in Figure 3.

To share information on the web without disputes, Description Logic (DL, formal knowledge representation language) can be used. DL is based on first-order logic to represent the knowledge of a particular domain and then the use of domain concepts (knowledge) to specify relationships among objects and individuals occurring in that domain.

Moreover, since the knowledge-representing systems answer the queries of a user in a reasonable time, DL can be used to describe the information of services as:

SrvDemand→ srv:Service∧(∃srv:has_Location.srvc:MedicalWard)∧(∃srvc: performs_Activity.srvc:CarbonDioxideMeasurement)∧(∃srvc:has_RemainingBatteryPower.srvc ≥ 80)∧(∃srvc:has_Reliability.srvc ≥ 95)∧(∃srvc:has_ResponseTime.srvc ≤ 1)

Following is the description logic notions of a few device services.

SrvOffer1→srvc:Service∧(∃srvc:has_Location.srvc:SurgicalWard∧ (∃srvc:performs_Activity.srvc:TemperatureMeasurement)∧(∃ srvc:has_RemainingBatteryPower.srvc = 51)∧(∃srvc:has_Reliability.srvc = 67)∧(∃srvc: has_ResponseTime.srvc = 5)
SrvOffer2→srvc:Service∧(∃srvc:has_Location.srvc:NICU)∧(∃srvc:performs_Activity.srvc:HumidityMeasurement)∧(∃srvc:has_RemainingBatteryPower.srvc = 65)∧(∃srvc:has_Reliability.srvc = 53)∧(∃srvc:has_ResponseTime.srvc = 8).
SrvOffer3→srvc:Service∧(∃srvc:has_Location.srvc:MedicalWard)∧(∃srvc:performs_Activity.srvc:CarbondioxideMeasurement)∧(∃srvc:has_RemainingBatteryPower.srvc = 76)∧(∃srvc:has_Reliability.srvc = 67)∧(∃srvc:has_ResponseTime.srvc = 3).

If the hospital administration wants to measure the level of carbon dioxide in the medical ward to take necessary actions if needed, they can use the services of devices installed on those premises. The services offered by different devices from different locations in a human-understandable format might be as given in Table 4. From Table 4, the last row, marked with Service Id SrvDemand, could be a demanding service by hospital administration, while rests are the services offered by different devices. Due to the non-availability of the publicly available data, we have used a random number generator to generate a dataset for 100 services. In order to get confidence that data is properly generated and represents the original dataset, several domain experts were consulted.

Upon consensus, a dataset was finalized, among which 10 services were selected to demonstrate the application of the proposed technique. The high-level detail of these services can be seen in Table 4. Moreover, the following attribute priorities were also decided in the meeting.

Context-Awareness Priority = 0.6; QoS Priority = 0.4; Location = 0.4; Upper Defined Value = 0.7; Lower Defined Value = 0.3; Activity = 0.3; Reliability = 0.2.

A query may consist of two types of concepts that either belong to the context-awareness or QoS. In the case of a concept belonging to the context-awareness, the similarity is judged through the mechanism defined in Section 3.1. For example, a user query defined in the last row of Table 4 has its first attribute of Activity since this attribute belongs to context-awareness; therefore, its closeness is judged through taxonomic relation as defined in Section 3.1.

While, in the case of QoS variables, users may define which values are preferable (higher values or lower values). For example, in the case of reliability attribute, higher values are preferable, while in the case of response time, lower values are preferable. The proposed system uses Equation (5) if higher values are required by the user in his/her request, while the system uses Equation (6) if lower values are required.

However, after submitting a request, the proposed system starts calculating the semantic similarity score (Equations (1)–(6)) of offered services (Table 5) and return category wise ordered list of services. Table 5 presents the computed score of these services through the proposed technique, while in Table 6 score of services is computed using the technique proposed by Bandara [44] (best work found in literature as it fulfills most of the requirements).

In Table 5, the score of Activity attribute of service Offer1 is zero because both attributes are disjoint, whereas the value of Activity attribute of service Offer7 is 0.3 because this score is calculated according to the rules defined in Section 3.1, by considering the semantic deviation.

Similarly, the value of Reliability attribute of service Offer1 is 0.941, calculated using Equation (5) after conformance metric matching rules for two QoS metrics as proposed in Equation (7). The scores for the rest of the services are calculated similarly. The sorted list of services is: SrvOffer3, SrvOffer7, SrvOffer10, SrvOffer9, SrvOffer5, SrvOffer6, SrvOffer1, SrvOffer4, SrvOffer8, and SrvOffer2 (given in Table 7).

In Table 6, scores of the same set of services as described in Table 5 are calculated by the technique proposed by Bandara et al. [44]. An ordered list of services is: SrvOffer3, SrvOffer10, SrvOffer7, SrvOffer5, SrvOffer6, SrvOffer9, SrvOffer2, SrvOffer4, SrvOffer8, and SrvOffer1.

### Expert Judgment

Most of the researchers conduct surveys to answer their research questions. Through a survey, respondents provide meaningful opinions, comments, and feedback based on their real-world observations. By analyzing these results, researchers can give the guidelines on how to address the issue instead of wasting resources on the issues having less or no importance by making sensible decisions. Therefore, we also conducted a survey. In this regard, we distributed a questionnaire among domain users and requested them to sort the services in ascending order. Details of their responses are given in Table 7.

Ordered list of services based on the score given in Table 7 is: SrvOffer3, SrvOffer7, SrvOffer10, SrvOffer1, SrvOffer2, SrvOffer9, SrvOffer5, SrvOffer6, SrvOffer4, and SrvOffer8.

In the above experiment, it was observed that by varying the wait of user priorities the final score of services also changes, but the order of services does not change. Whereas this is not the case with the survey, in a survey by changing priorities, the results of the ordered list are changed altogether.

## 5. Results and Discussion

In the above section, service score computation with the help of three different methods (proposed, best from literature and human) is performed. In this section, an explanation is given on how the proposed method is better than existing techniques by comparing the results with earlier findings and the possible reasons behind the betterment of the proposed technique.

By looking at the service list of Table 5, Super match list = [ᴓ]; Good partial match list = [SrvOffer3, SrvOffer7, SrvOffer10]; Partial match list = [SrvOffer9, SrvOffer5, SrvOffer6, SrvOffer1, SrvOffer4, SrvOffer8, SrvOffer2]; Fail match list = [ᴓ]. Similarly, from the data of services of Table 6, none exactly match nor totally fails to fulfill the user demand, while SrvOffer2, SrvOffer4, SrvOffer5, SrvOffer6, SrvOffer8, SrvOffer9 meet most of the user requirements whereas SrvOffer2, SrvOffer4, SrvOffer8, SrvOffer1 fulfill the least set of requirements. In the same fashion, if we look at the data collected from human through a questionnaire (Table 7), only SrvOffer3 services fulfill all of the user-defined requirements. On the other hand, services SrvOffer4, SrvOffer8 do not fulfill any of the requirements as reported by the experts. However, SrvOffer7, SrvOffer10 are the services that meet most of the requirements after service SrvOffer3, whereas SrvOffer1, SrvOffer2, SrvOffer9, SrvOffer5, SrvOffer6 meet the least requirements in descending order.

Similarly, if we look at the top five results of all three techniques by making a sorted list of each, we can observe the resemblance between the proposed technique and the human services list. According to the observation, the order of the first three services of both techniques (our proposed and the human) is the same. However, the technique proposed by Bandara et al. [44] also produces the same set of the first three services, but the order is different. This means that the proposed technique can produce more accurate results than the technique proposed by Bandara et al. [44]. Moreover, it can also produce results according to human perception. For example, a set of three services produced by our technique is SrvOffer3, SrvOffer7, SrvOffer10, which is the same set of services produced by humans while the order is changed (SrvOffer3, SrvOffer10, SrvOffer7) by Bandara et al. [44] approach. Bandara et al. [44] approach suggests that service SrvOffer10 meets second-highest requirements of users that is actually not right because it is confirmed by the human response.

**Figure 4:**
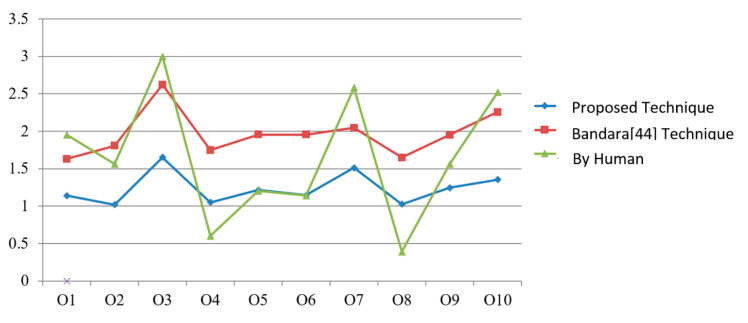
Comparison of service scores of different techniques.

In order to understand the causes of variation in score resulting in a different ordered list, we have drawn a line graph, which is presented in Figure 4.

X-axis represents the services while service scores are plotted on *Y*-axis. Blue line of the graph represents the proposed approach, green for humans, and the red line represents the Bandara et al. [44] approach. By analyzing results, it is now clear that the variation in service scores is due to the consideration of a number of factors like the consideration of user assigned priorities (individual concept as well as group), value type direction, along with the consideration of rich context-awareness.

### 5.1. Significance Test

In the above section, it is concluded that the proposed technique can produce results near to human understanding. In this section, we are using statistical methods to explain the data and relationships in a meaningful way. In this regard, an independent samples *t* test experiment is conducted. Test statistics are given in Table 8.

However, before executing the test, the hypothesis for the test is: The consideration of prioritization, grouping, value direction, and the context-aware knowledge of devices during service discovery can produce accurate results.

The Sig. (2-Tailed) value of the proposed method is 0 (Table 9); therefore, it is concluded that there is a statistically significant difference between the techniques which considers prioritization, grouping priorities, value direction, and context knowledge during the services discovery process and the technique that does not consider all these parameters. Moreover, to measure uncertainty, the standard deviation can be used because it tells the data distribution information around mean value. In this regard, from Table 8, one can observe the standard deviation of the proposed technique is 0.212, which is less than the standard deviation (0.352) of the technique proposed by Bandara et al. [44]. This fact indicates that the proposed technique produces more stable results with respect to the Bandara et al. [44] technique.

Similarly, in order to prove it statistically that the proposed technique can produce results as per human understanding, null and the alternate hypotheses are designed as:

**Hypothesis 0 (H0).**  *A technique considering prioritization, grouping, value direction, quality of service, and rich knowledge of context-awareness during the services discovery process still cannot produce results according to human understanding.*

**Hypothesis 1 (H1).**  *A technique considering prioritization, grouping, value direction, quality of service, and rich knowledge of context-awareness during the services discovery process can produce results according to human understanding.*

Test statistics are given in Table 10 while the Sig. (2-Tailed) value is 0.001 (Table 11); therefore, it is concluded that the null hypothesis is rejected and an alternate hypothesis is accepted, which states that the proposed technique can produce results according to human understanding. By analyzing Table 7 and Table 8, one can observe the big difference between standard deviations of the proposed technique and human understanding. The possible reason behind this difference could be, since, survey respondents were asked to rank services between 1 and 10 while considering the priorities.

Whereas the proposed technique computes the score of individual service attributes between 0 and 1 and multiplies, the individual service attributes score with the priority assigned to it. Therefore, the score of standard deviation is reduced as compared to the human participants.

### 5.2. Performance Evaluation

**Figure 5:**
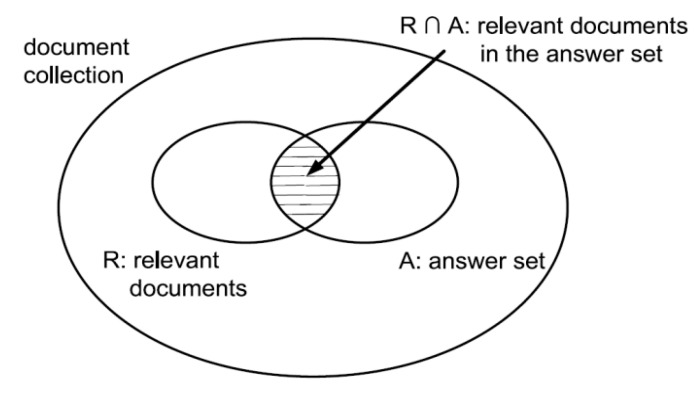
Performance evaluation measures.

Information retrieval has been developed as a high empirical discipline requiring a careful and complete evaluation to show the performance of techniques on the representative service collections. The standard approach for measuring the performance of such techniques revolves around the notion of relevant and non-relevant service. A service is said to be relevant if it addresses the requirements of the user; not only as it just happens to contain all of the concepts in the query. In order to evaluate a technique, an explicit expression is required for the judgment of returned results as relevant or non-relevant. In this regard, the two most frequent and basic measures (precision and recall) for judging the performance [64] is used. These measures are presented in Figure 5, where, I is an information request, R is the set of relevant documents for I, A is the answer set for I, and finally R ∩ A is the intersection of the sets R and A.

Precision (Equation (10)) is the fraction of the retrieved services that are relevant (Number of retrieved resources that are relevant/Number of retrieved resources).

(10) $$Precision\;=\;\frac{\left|\;R\;\cap \;A\;\right|}{\left|\;A\;\right|}$$

Recall (Equation (11)) is the fraction of the relevant services that has been retrieved (Number of retrieved resources that are relevant/Number of relevant resources).

(11) $$Recall\;=\;\frac{\left|\;R\;\cap \;A\;\right|}{\left|\;R\;\right|}\;$$

The F1 Metric (Equation (12)) attempts to combine Precision and Recall into a single value for comparison purposes. It is the weighted harmonic mean of precision and recall used to gain a more balanced view of performance.

(12) $$F1=2*\frac{Precision*Recall}{Precision+Recall}\;$$

**Figure 6:**
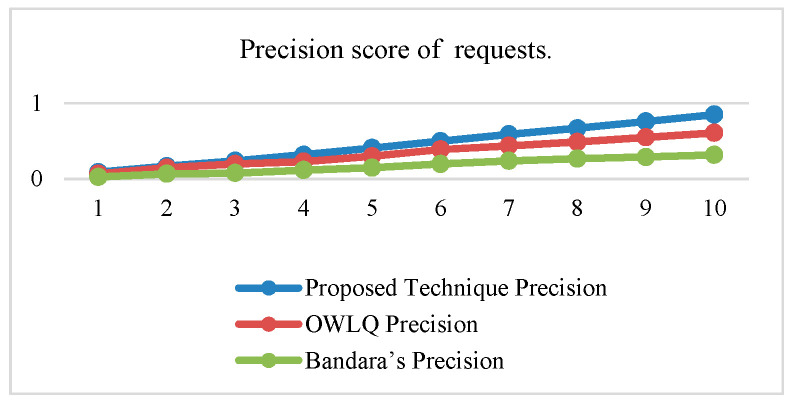
Precision of service requests.

**Figure 7:**
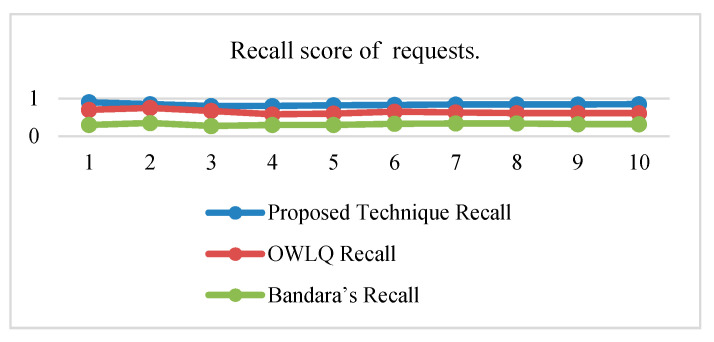
Recall score of services.

**Figure 8:**
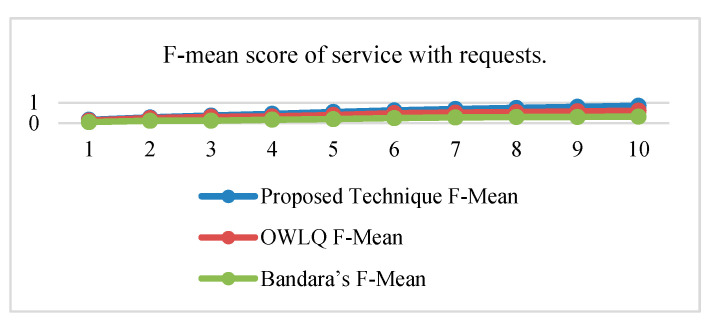
F-mean score of services.

Precision, recall, and F-measure values of proposed OWLQ [45] and Bandara’s Techniques [44] according to the Equations (10)–(12) are presented in Table 12. The precision, recall, and F-measure values given in Table 12 are plotted in Figure 6, Figure 7 and Figure 8. The average precision and recall values of the proposed technique are 0.46 and 0.84, respectively, whereas these values for the OWLQ framework are 0.34 and 0.64. It is observed that the proposed technique has better precision and recall values as compared to the OWLQ framework, which is a 26% increase in precision and a 23.8% increase in recall. The F-measure (weighted harmonic mean of precision and recall) for the proposed technique is 0.164 as opposed to the OWLQ framework, which has 0.127 for the first group. The average F-measure of proposed technique is 0.55, while the average F-measure of OWLQ framework is 0.42, which is 23.64% less to the proposed technique.

Similarly, the precision, recall, and F-measure values for both of the frameworks (proposed and Bandara’s) are given in Table 12 and plotted in Figure 6, Figure 7 and Figure 8. The average precision and recall values of the proposed technique are 0.46 and 0.84, respectively, whereas these values for the Bandara’s framework are 0.18 and 0.32. It is observed that the proposed technique has better precision and recall values as compared to Bandara’s framework, which is a 61% increase in the precision and a 62% increase in recall.

The F-measure (weighted harmonic mean of precision and recall) for the proposed technique is 0.164 as opposed to the Bandara’s framework, which has 0.055 for the first group. The averaged F-measure of the proposed technique is 0.55, while the averaged F-measure of Bandara’s framework is 0.21, which is 61.82% less to the proposed technique.

From the above analysis, it is concluded that the proposed technique has better service discovery performance compared to the OWLQ and Bandara’s frameworks. It is also observed that better performance is due to the consideration of comparison, context-awareness, grouping, group priorities, and value type direction.

## 6. Conclusions

Smart systems operating in distributed computing environments are much different from traditional dynamic systems as they may interconnect hundreds of IoT nodes. This massive interconnection and the advanced microprocessor technology had made smart systems competitive and complex. The complexity in terms of heterogeneity restricts different devices to share integrated knowledge effectively with each other. In order to address the issue of heterogeneity of devices effectively, SOA methodology can be used, where every device can participate in the activity of production, integration, and transferring data between them regardless of device complexity. SOA relies on web services and semantic web, the advanced form of web, handles the complexity with the help of ontologies. Whereas, techniques like case-based reasoning, probabilistic graphical models, and artificial neural networks cannot be used to model incomplete domain knowledge effectively. Therefore, the proposed logic and semantic-based hybrid framework can handle the issue of uncertainty for uniform knowledge sharing in distributed computing environments. Evaluation results confirm the correctness of the proposed framework. In the future, the impact of the network, the issues of scalability, and fault tolerance in the framework will be investigated.

## Figures and Tables

**Table 1:** Smart system’s general criteria requirements.

Requirement	Description
Grouping	In order to apply group rules on requirements, it is necessary to group similar nature of requirements. The groups are defined based on the objectives. In this study, two groups of requirements are defined.
Value type direction	By using value type direction, the user can instruct the system on how to process the requirement. For example, in the reliability case, a higher value might be preferable for the user, in this case, the value type direction should be positive. However, in the case of response time, the value type direction should be opposite.
Prioritization	The system should allow the user to assign priorities to each requirement. In this case, the automatic selection from candidate services may become more accurate to meet the user needs.
Group prioritization	The system should allow users to assign priority to each group along with the assignment of priorities to each requirement. For example, users should be allowed to assign priority to each group (Context-Awareness and QoS) cumulating to 1 (between 0 to 1).
Categorization	The system should categorize each device service according to its fulfillment of user-defined criteria. This is an important requirement because if a device suddenly stops its services, the system may automatically pick the second service to use from the list meeting most of the user-defined requirements.
Approximate matching	It is the measure of the degree of matching between the use request and the device services. This requirement is particularly important in the scenario where if exact services are not found, the service requester might be interested to use the service meeting most of the user-defined requirements.
Metric matching	QoS metric measures the QoS value of a service. Hence, it is necessary that the explicit description QoS metric must be defined in the discovery framework.

**Table 2:** Smart system’s specific requirements.

Requirement	Description
Location	Smart systems can automatically track the location of objects in real-time in order to use their services.
Activity	Activity is a measurable amount of action that any device can perform. For example, a device can measure temperature or humidity in the room, etc.
Timeliness	It is a time required to access a service and the use of information. It can be measured as the time of request submission and the use of device services.
Interoperability	Interoperability means that devices from different operating platforms and speaking different languages can operate together. It is an essential property of smart objects.
Robustness	It is the ability of the system to provide functionality even if some device services become unavailable. In dynamic systems, the appearance and disappearance of services are not unusual.
Adaptability	Adaptability means that the system can detect if new devices services become available, and recompose with better services to perform well.

**Table 3:** Comparison among existing techniques for service discovery.

Techniques	Criteria Requirements													
	General Purpose Requirements								Dynamic Requirements					
	Grouping	Prioritization	Value Type Direction	Group Prioritization	Categorization	Approximate Matching	Metric Matching	Activity	Resources	Location	Timeliness	Interoperability	Robustness	Adaptability
Baocai et al. [23]		√										√		
Ma et al. [28]										√			√	√
Chua et al. [29]		√					√				√			
Ayadi et al. [30]				√						√				√
Zhao et al. [31]	√					√						√		
Li et al. [32]		√		√	√				√					
Zhu et al. [34]		√					√						√	
Arabshian et al. [36]			√					√				√		
Santos et al. [40]	√				√					√				
Bandara_A. et al. [44]		√			√	√		√	√			√	√	√
Kritikos and Plexousakis [45]	√				√	√	√		√			√	√	√
Guo et al. [46]							√							
Suraci et al. [47]		√							√					√
Gu et al. [48]	√					√				√			√	
Liang et al. [49]		√	√			√			√					√
Daniele et al. [50]				√			√			√				
Chen et al. [51]									√		√	√		
Mahmoodpour et al. [52]					√				√		√	√		
Sobral et al. [53]	√		√		√				√	√	√	√		

**Table 4:** High-level description of service offered by devices.

Service ID	Activity	Location	Battery Power	Reliability	Response Time
SrvOffer1	Temperature Measurement	Surgical Ward	51	67	5
SrvOffer2	Humidity Measurement	NICU	65	53	8
SrvOffer3	Carbon dioxide Measurement	Medical Ward	76	67	3
SrvOffer4	Light Level Measurement	NICU	59	75	5
SrvOffer5	Temperature Measurement	Room	79	61	4
SrvOffer6	Humidity Measurement	Laboratory	75	75	6
SrvOffer7	Carbon dioxide Measurement	Surgical Ward	61	76	2
SrvOffer8	Light Level Measurement	Laboratory	53	69	4
SrvOffer9	Temperature Measurement	PICU	76	82	3
SrvOffer10	Humidity Measurement	Medical Ward	67	79	5
SrvDemand	Carbon dioxide Measurement	Medical Ward	80	95	1

**Table 5:** Services score computed through the proposed technique.

Service ID	Activity	Location	Battery Power	Reliability	Response Time	Total Score
SrvOffer1	0.000	0.264	0.638	0.941	0.556	1.140
SrvOffer2	0.000	0.000	0.813	0.912	0.417	1.020
SrvOffer3	0.300	0.400	0.950	0.941	0.714	1.652
SrvOffer4	0.000	0.000	0.738	0.958	0.556	1.049
SrvOffer5	0.000	0.000	0.988	0.928	0.625	1.214
SrvOffer6	0.000	0.000	0.938	0.958	0.500	1.146
SrvOffer7	0.300	0.264	0.763	0.960	0.833	1.513
SrvOffer8	0.000	0.000	0.663	0.945	0.625	1.026
SrvOffer9	0.000	0.000	0.950	0.973	0.714	1.245
SrvOffer10	0.000	0.400	0.838	0.966	0.556	1.352
SrvDemand	0.300	0.400	1.000	1.000	1.000	1.820

**Table 6:** Scores calculated through Bandara approach [14].

Service ID	Activity	Location	Battery Power	Reliability	Response Time	Total Score
SrvOffer1	0.00	0.00	0.638	0.941	0.05	1.629
SrvOffer2	0.00	0.00	0.813	0.912	0.08	1.805
SrvOffer3	0.30	0.40	0.950	0.941	0.03	2.621
SrvOffer4	0.00	0.00	0.738	0.958	0.05	1.746
SrvOffer5	0.00	0.00	0.988	0.928	0.04	1.956
SrvOffer6	0.00	0.00	0.938	0.958	0.06	1.956
SrvOffer7	0.30	0.00	0.763	0.960	0.02	2.043
SrvOffer8	0.00	0.00	0.663	0.945	0.04	1.648
SrvOffer9	0.00	0.00	0.950	0.973	0.03	1.953
SrvOffer10	0.00	0.40	0.838	0.966	0.05	2.254
SrvDemand	0.30	0.40	1.000	1.000	0.01	2.710

**Table 7:** Human response on services.

Service ID	Prsn1	Prsn2	Prsn3	Prsn4	Prsn5	Prsn6	Prsn7	Prsn8	Prsn9	Prsn10	Total Score
SrvOffer1	4	6	4	4	5	4	5	4	4	5	4.5
SrvOffer2	6	5	7	7	4	5	6	7	7	4	5.8
SrvOffer3	1	1	1	1	1	1	1	1	1	1	1.0
SrvOffer4	7	9	10	9	9	10	9	8	10	9	9.0
SrvOffer5	8	8	6	6	8	7	7	6	6	8	7.0
SrvOffer6	9	4	8	8	7	8	4	9	8	7	7.2
SrvOffer7	2	2	3	3	2	2	2	3	3	2	2.5
SrvOffer8	10	10	9	10	10	9	10	10	9	10	9.7
SrvOffer9	5	7	5	5	6	6	8	5	5	6	5.8
SrvOffer10	3	3	2	2	3	3	3	2	2	3	2.5

**Table 8:** Test statistics of *t*-test (Group Statistics).

Technique	N	Mean	Std. Deviation	Std. Error Mean
Score Bandara Technique	10	3.616	0.352	0.111
Proposed Technique	10	1.235	0.212	0.067

**Table 9:** Test statistics with the best approach.

	Levene’s Test for Equality of Variance		*t*-Test for Equality of Means				
						95% Confidence Interval of the Difference	
	F	Sig.	*t*	df	Sig.(2-tailed)	Lower	Upper
Equal variances	6.627	0.019	18.30	18	0.000	2.107	2.653
Unequal variances			18.30	14.79	0.000	2.103	2.657

**Table 10:** Test statistics of *t*-test (Group Statistics).

Technique	N	Mean	Std. Deviation	Std. Error Mean
Score Proposed Technique	10	1.235	0.212	0.067
Human Understanding	10	5.500	2.876	0.909

**Table 11:** *t*-test statistics with survey results.

	Levene’s Test for Equality of Variance		*t*-Test for Equality of Means				
						95% Confidence Interval of the Difference	
	F	Sig.	*t*	df	Sig.(2-tailed)	Lower	Upper
Equal variances	18.92	0.0	−4.68	18	0.000	−6.18	−2.35
Unequal variances			−4.68	9.098	0.001	−6.32	−2.20

**Table 12:** Precision, recall, and f-measure of proposed, OWLQ and Bandara’s techniques.

Top % Services	Proposed Technique	OWLQ	Bandara’s	Proposed Technique	OWLQ	Bandara’s	Proposed Technique	OWLQ	Bandara’s
	Precision	Precision	Precision	Recall	Recall	Recall	F-Mean	F-Mean	F-Mean
10	0.09	0.07	0.03	0.90	0.70	0.30	0.164	0.127	0.055
20	0.17	0.15	0.07	0.85	0.75	0.35	0.283	0.25	0.117
30	0.24	0.20	0.08	0.80	0.67	0.27	0.369	0.308	0.123
40	0.32	0.23	0.12	0.80	0.58	0.30	0.457	0.329	0.171
50	0.41	0.30	0.15	0.82	0.60	0.30	0.547	0.400	0.200
60	0.50	0.39	0.20	0.83	0.65	0.33	0.624	0.488	0.249
70	0.59	0.44	0.24	0.84	0.63	0.34	0.693	0.518	0.281
80	0.67	0.49	0.27	0.84	0.61	0.34	0.745	0.543	0.301
90	0.76	0.55	0.29	0.84	0.61	0.32	0.798	0.578	0.304
100	0.85	0.61	0.32	0.85	0.61	0.32	0.850	0.610	0.320
